# Machine learning-based integration develops an immune-derived signature for diagnosing high-altitude pulmonary hypertension

**DOI:** 10.3389/fmed.2025.1603140

**Published:** 2025-09-02

**Authors:** Dan Yang, Qian Li, Feng Yang, Rui Wang, Peng Jiang, Jialin Wu, Xi Yang, Yixuan Huang, Yuqiang Liu, Shishang Wang, Junqiang Gou, Zhangfeng Sun, Junjie Ma, Yanhui Qin, Wu Li, Dongfeng Yin

**Affiliations:** ^1^General Hospital of Xinjiang Military Command, Urumqi, China; ^2^Xinjiang Medical University, Urumqi, China; ^3^No.951 Hospital of PLA, Korla, China; ^4^Shihezi University, Shihezi, China

**Keywords:** high-altitude pulmonary hypertension, single-cell RNA sequencing, multi-omics integration, machine learning, non-invasive diagnosis

## Abstract

**Background:**

High-altitude pulmonary hypertension (HAPH) is a common disease in high-altitude regions where implementation of gold-standard diagnostic methods remains logistically challenging.

**Methods:**

In the retrospective analysis, we employed an integrative multi-omics approach combining single-cell RNA sequencing (scRNA-seq, *n* = 10), bulk RNA sequencing (RNA-seq, *n* = 126), and proteomic profiling (*n* = 42) to characterize immune microenvironment remodeling in HAPH. Subsequently, we established a machine learning-based diagnostic model. The HAPH-associated signatures were finally validated by Quantitative PCR.

**Results:**

Through scRNA-seq analysis utilizing Ro/e and contribution scoring analysis, we first demonstrated the pivotal role of myeloid lineages in HAPH pathogenesis. Pseudotime trajectory analysis of the myeloid subsets further revealed 2,615 differentially expressed genes (DEGs) associated with HAPH progression. We also identified 144 and 77 DEGs from bulk RNA-seq and proteomic data between HAPH and control groups, respectively. Finally, 22 candidate biomarkers were screened by muti-omics analysis. These genes were further refined through ensemble machine learning algorithms. Evaluation of 113 algorithm combinations revealed that a six-gene random forest (RF) model (HEMGN, HBG2, MYL9, ANK1, UBE2O, RBPMS2) achieved optimal diagnostic accuracy, with an area under the curve (AUC) of 0.995 in the training cohort (*n* = 55) and 0.773 in external validation cohorts (*n* = 71). Quantitative PCR validated significant overexpression of these biomarkers in HAPH compared to controls (*p* < 0.05).

**Conclusion:**

Our findings propose the minimally invasive blood-derived immune signature for HAPH diagnosis, providing a practical framework for early detection in resource-constrained high-altitude populations.

## Introduction

High-altitude pulmonary hypertension (HAPH) is a progressive cardiopulmonary disorder caused by hypoxic exposure. Epidemiological data indicate that approximately 140 million people permanently reside above 2,500 m, with an additional 40 million annual transient visitors to these high-altitude regions (1, 2). Observational studies demonstrate that HAPH prevalence among permanent high-altitude dwellers ranges from 6 to 35% (2). The initial clinical manifestations of HAPH are frequently nonspecific, resulting in delayed diagnosis and compromised therapeutic efficacy. These diagnostic challenges highlight the urgent need for early detection of HAPH, which could significantly reduce mortality rates and improve prognosis. Thus, developing evidence-based diagnostic protocols remains a critical unmet need in altitude medicine.

The diagnosis of HAPH necessitates right heart catheterization (RHC)-derived mean pulmonary arterial pressure (mPAP) measurements, with a diagnostic threshold established at >25 mmHg (3). Although RHC remains the gold standard, its invasive nature and dependence on specialized medical infrastructure critically constrain its implementation in high-altitude clinical settings. Novel non-invasive approaches integrating machine learning with echocardiographic and cardiac MRI techniques exhibit diagnostic promise; however, persistent technological challenges and resource disparities hinder their widespread adoption in mountainous regions (4, 5). In contrast, blood-based biomarker assays address these diagnostic limitations through minimally invasive sampling and standardized protocols, offering particular advantages in resource-limited environments (6). For instance, dried blood spot microsampling techniques—requiring only capillary blood collection—preserve sample integrity under hypobaric conditions, establishing a viable platform for altitude-adapted diagnostic frameworks (7). Concurrently, advancements in high-throughput omics profiling have uncovered molecular mechanisms underlying pulmonary hypertension pathogenesis (8, 9). Large-scale meta-analyses of circulatory biomarkers reveal conserved immunopathological dysregulation patterns aligned with tissue-level disease manifestations (8, 10). Validation cohorts further substantiate the utility of peripheral blood immune signatures as multidimensional classifiers for polygenic diseases (11, 12). Together, these developments underscore the potential of integrated liquid biopsy biomarker panels to enable molecular-guided stratification in precision HAPH management.

Therefore, in this study, we aimed to establish a robust diagnostic signature for HAPH. Employing ensemble machine learning with leave-one-out cross-validation (LOOCV) based on multi-omics data (including single-cell RNA sequencing, bulk transcriptomic profiles, and proteomic data), we constructed and validated the signature. The results could provide a framework for personalized therapeutic strategies while enhancing prognostic prediction in HAPH clinical practice, ultimately improving patient outcomes.

## Materials and methods

### Collecting HAPH patients and samples

The peripheral blood mononuclear cell (PBMC) samples utilized in this retrospective study were obtained from residual or discarded clinical specimens collected during routine diagnostic and therapeutic procedures from the General Hospital of Xinjiang Military Command with approval from the Institutional Review Board (2020RR0618). Written informed consent was obtained from all participants. Comprehensive clinical characteristics were presented in Supplementary Table S1. The single-cell RNA sequencing (scRNA-seq) cohort comprised 5 patients with high-altitude pulmonary hypertension (HAPH) and 5 matched healthy controls. We employed the SCOPIT (V1.1.4) to perform power analysis to determine the number of cells required per sample (Supplementary Figure S1; Supplementary Table S2), following established methodologies (13, 14). For bulk RNA sequencing, we assembled a composite cohort of 56 cases and 70 controls by integrating in-house samples with other group available dataset, which served as independent validation cohorts. The training and validation datasets were sourced from distinct institutions, with no overlap between them. The clinical baseline characteristics of the train and validation cohorts were summarized in Supplementary Table S3. Proteomic profiling was performed on PBMC samples from 18 HAPH patients and 24 healthy controls.

### scRNA-seq of HAPH and data processing

The PBMC samples were processed following an optimized protocol derived from established methodology (15). Cell viability was quantified (>90%) before loading suspensions onto a Chromium Single-Cell Controller (10 × Genomics) for library preparation. Sequencing reads were aligned to the GRCh38 reference genome using CellRanger (v8.0.0). Downstream analyses incorporated SCTransform normalization and Harmony integration via the Seurat (v4.0.2) (16) in R (v4.3.3) (17). Quality control retained cells expressing 500–4,000 genes with mitochondrial gene content <15%. Cell-type identification utilized canonical marker genes. Pseudobulk differential expression analysis was conducted using glmGamPoi (v1.12.2) (18), with significance criteria of |log2 (fold change)| > 0.5 and adjusted *p* < 0.05.

### Identification of hub subsets in HAPH immune microenvironment

We employed the observed to predicted cell number (Ro/e) ratio and contribution scoring to identify hub immune cell subsets in HAPH (19). To quantify cluster-specific group preferences, we calculated the Ro/e ratio for each cell cluster across experimental groups using a validated methodology. Predicted cell numbers for cluster-group combinations were derived through chi-square testing. Contribution scores of cellular subgroups were computed following an established computational framework (20). Specifically, signature genes for each subset were defined as the top 100 differentially expressed genes (DEGs) between HAPH and control groups. These scores integrate both quantitative changes in cell populations and expression-level alterations of signature genes during disease progression. Final cluster contributions were determined by averaging the fold-change scores of all signature genes within each cellular subgroup.

### Pseudotime trajectory analysis of myeloid cells

To elucidate molecular mechanisms underlying HAPH progression, we conducted pseudotime trajectory reconstruction of myeloid cell differentiation using Monocle2 (v2.18.0) (21). The differentGeneTest function identified differentially expressed genes (DEGs) correlated with pathological transition from normal vasculature to HAPH. We applied the discriminative dimensionality reduction via learning a tree (DDRTree) algorithm for trajectory inference, followed by cellular ordering in reduced dimensional space. Pseudotemporal analysis revealed temporally upregulated HAPH-progression genes (adjusted *p* < 0.05), implicating their potential mechanistic contributions to disease advancement.

### Identifying the genes related to HAPH by bulk RNA-seq

To identify genes associated with HAPH, we conducted bulk RNA-seq analysis PBMC samples from 56 HAPH patients and 70 healthy controls. Total RNA extraction was performed using the TRNzol Total RNA Extraction Reagent Kit. Samples meeting quality control thresholds [RNA integrity number (RIN) > 7.0 and 28S/18S ratio ≥ 0.7] were selected for library preparation. Sequencing was conducted on the Illumina HiSeq PE150 platform with 150-bp paired-end reads. Raw sequencing reads were aligned to the GRCh38 human reference genome using STAR software (v2.7.2a) (22), with subsequent gene-level quantification performed through htseq-count (v2.05) (23). The ComBat algorithm was used to eliminate batch effects. Differential gene expression analysis was conducted using DESeq2 (v1.40.2) (24), with statistically significant genes identified using thresholds of |log2 (fold change) | > 0.5 and adjusted *p* < 0.05 (25, 26).

### Identifying the genes related to HAPH by proteomics

To identify potential biomarkers associated with HAPH, we performed comparative proteomic analysis of PBMC samples obtained from 18 HAPH patients and 24 healthy controls. Cellular proteins were extracted using a standardized lysis buffer according to established protocols. Subsequent proteomic profiling was conducted using liquid chromatography–tandem mass spectrometry (LC–MS/MS). Raw data from data-dependent acquisition (DDA) experiments were analyzed with MaxQuant software (v1.5.3.30) (27) against the Human reference proteome (UniProtKB; 46,570 sequences). We generated a spectral library in Spectronaut with the following parameters: trypsin digestion; minimum peptide length of 7 amino acids; variable modifications including methionine oxidation and N-terminal acetylation; fixed carbamidomethylation of cysteine residues; and a peptide-spectrum match (PSM) false discovery rate (FDR) ≤ 1%. Remaining parameters retained default configurations. For data-independent acquisition (DIA) analysis, Spectronaut executed spectral deconvolution using the preconstructed library and implemented quality control through the mProphet algorithm, yielding high-confidence quantitative profiles. MSstats software (v4.8.7) was employed to reduce batch effects (28). Differential protein expression between HAPH and control groups was statistically evaluated using MSstats (v4.14.2) (29), with significance thresholds established at *p* < 0.05 and |log2 (fold change)| ≥ 0.5. The overlapping candidate genes identified from scRNA-seq, bulk RNA-seq, and proteomic data were designated as HAPH signature genes.

### Signature generated from machine learning-based integrative approaches

To establish a consensus HAPH prognostic signature exhibiting robust predictive accuracy and stability, we systematically integrated 12 machine learning algorithms encompassing 113 methodological combinations (30, 31). The signature development protocol consisted of four key phases: (a) systematic application of 113 algorithm combinations to construct predictive models using a leave-one-out cross-validation (LOOCV) framework; (b) comprehensive validation of all models in the independent dataset; and (c) quantitative evaluation using Harrell’s concordance index (C-index), with model selection based on maximal average C-index performance across validation cohorts; (d) The Hosmer–Lemeshow test and Brier score were computed to assess the calibration of the model.

### Validation of HAPH-associated signatures

To validate the HAPH signature, we acquired distinct tissue samples from the original sequencing cohort for Quantitative real-time PCR (qPCR) validation. Total RNA was isolated using the RNA Extraction Kit, followed by reverse transcription into cDNA using the cDNA Synthesis Kit. qPCR analysis was performed in six technical replicates using SYBR Green Master Mix on a CFX Connect Real-Time PCR Detection System (Bio-Rad Laboratories, United States). Gene-specific primers (Supplementary Table S4) were used in 20 μL reaction volumes with standardized cycling parameters: initial denaturation at 95°C for 3 min, followed by 40 cycles of 95°C for 10 s and 60°C for 30 s. The GAPDH was employed as an endogenous control for normalization of mRNA expression levels. Relative quantification was calculated using the comparative threshold cycle (2^^−ΔΔCt^) method (26).

### Statistical analysis

All statistical analyses were conducted using R (v4.3.3). Prior to parametric testing, data were assessed for normality using the Shapiro–Wilk test and homogeneity of variance using Levene’s test. For group comparisons meeting these assumptions, unpaired two-tailed Student’s *t*-tests were employed. Statistical significance was defined as *p* < 0.05 for all analyses.

## Results

### Identification of the hub subsets in HAPH by scRNA-seq

**Figure 1:**
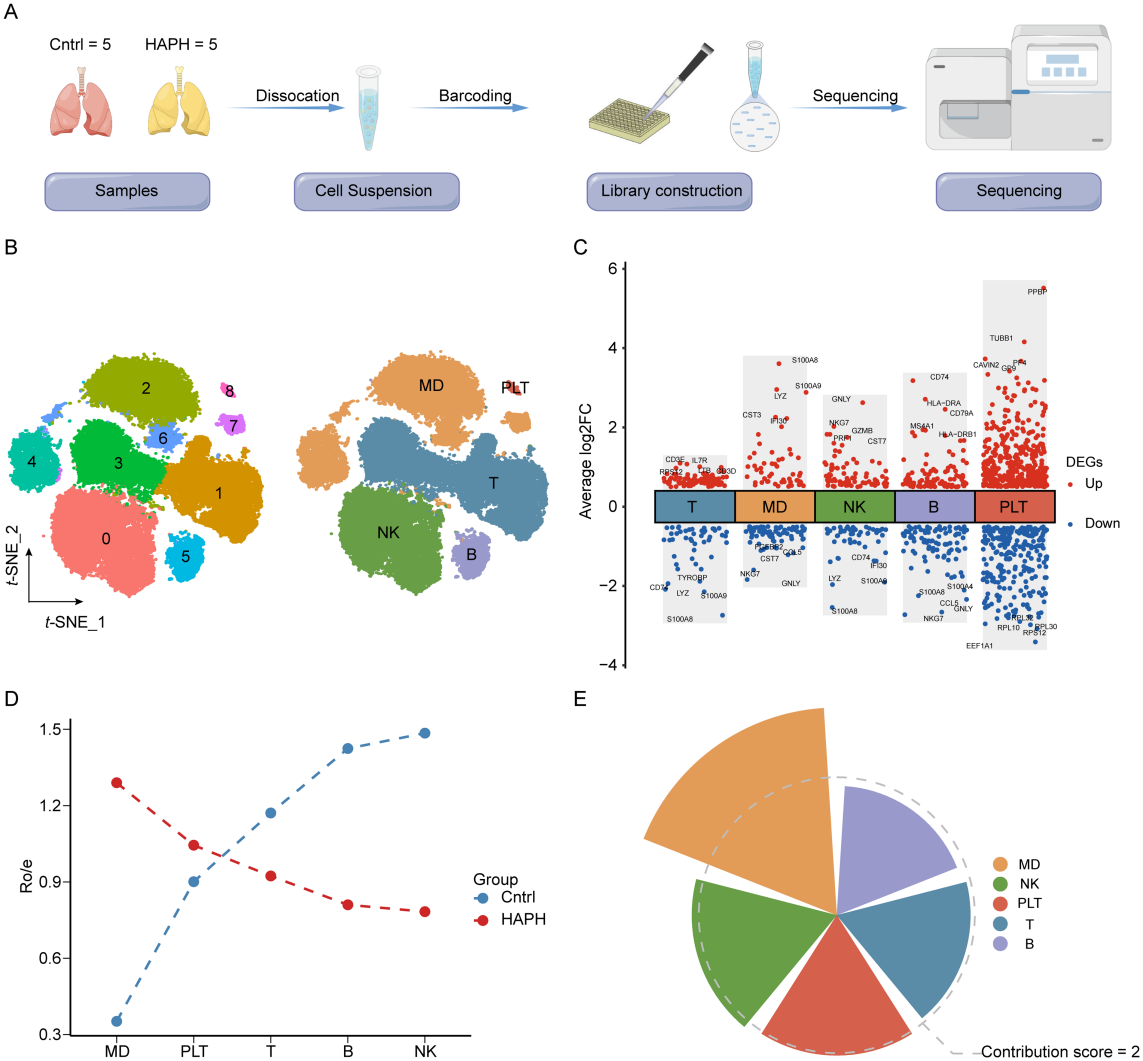
Single-cell RNA-seq profiling reveals immunological heterogeneity in the HAPH microenvironment. **(A)** Schematic workflow of sample collection and single-cell transcriptomic analysis procedures. **(B)** Dual-panel t-SNE visualization (left: cluster identity; right: annotated cell types) of 56,058 cells from 10 specimens (5 Control vs. 5 HAPH). **(C)** Volcano plot identifying differentially expressed genes (DEGs) across clusters. Significantly upregulated (red; FDR-adjusted *p* < 0.05) and downregulated (blue; FDR < 0.05) genes are demarcated, with non-significant transcripts in black. **(D)** Observed-to-expected (Ro/E) ratio quantification of cellular composition differences between HAPH and Control groups. **(E)** Radial plot visualizing disease-associated subsets contributions, where radial length encodes functional impact magnitude.

Our single-cell transcriptomic atlas comprising 56,058 single cells from 5 HAPH patients and 5 healthy controls (Cntrl) (Figure 1A) systematically mapped disease-associated immune microenvironment. Following SCTransform normalization and Harmony integration to mitigate batch effects, unsupervised clustering of harmonized data identified 15 distinct clusters (Figure 1B). Canonical marker expression analysis (Figure 1C) resolved five hematopoietic lineages: T lymphocytes (CD3D+), myeloid cells (LYZ+), natural killer cells (NKG7+), B lymphocytes (MS4A1+), and platelets (PPBP+). The observed to predicted cell number (Ro/e) ratio revealed significant myeloid compartment expansion in HAPH (Ro/e = 1.26) with concomitant NK cell depletion (Ro/e = 0.89) compared to Cntrl (Figure 1D). Applying a computational framework to quantify cellular pathogenic potential, we identified myeloid subpopulations as dominant contributors (contribution score = 2.86), implicating their functional centrality in HAPH pathophysiology.

### Trajectory analysis revealed the HPAH-associated genes

**Figure 2:**
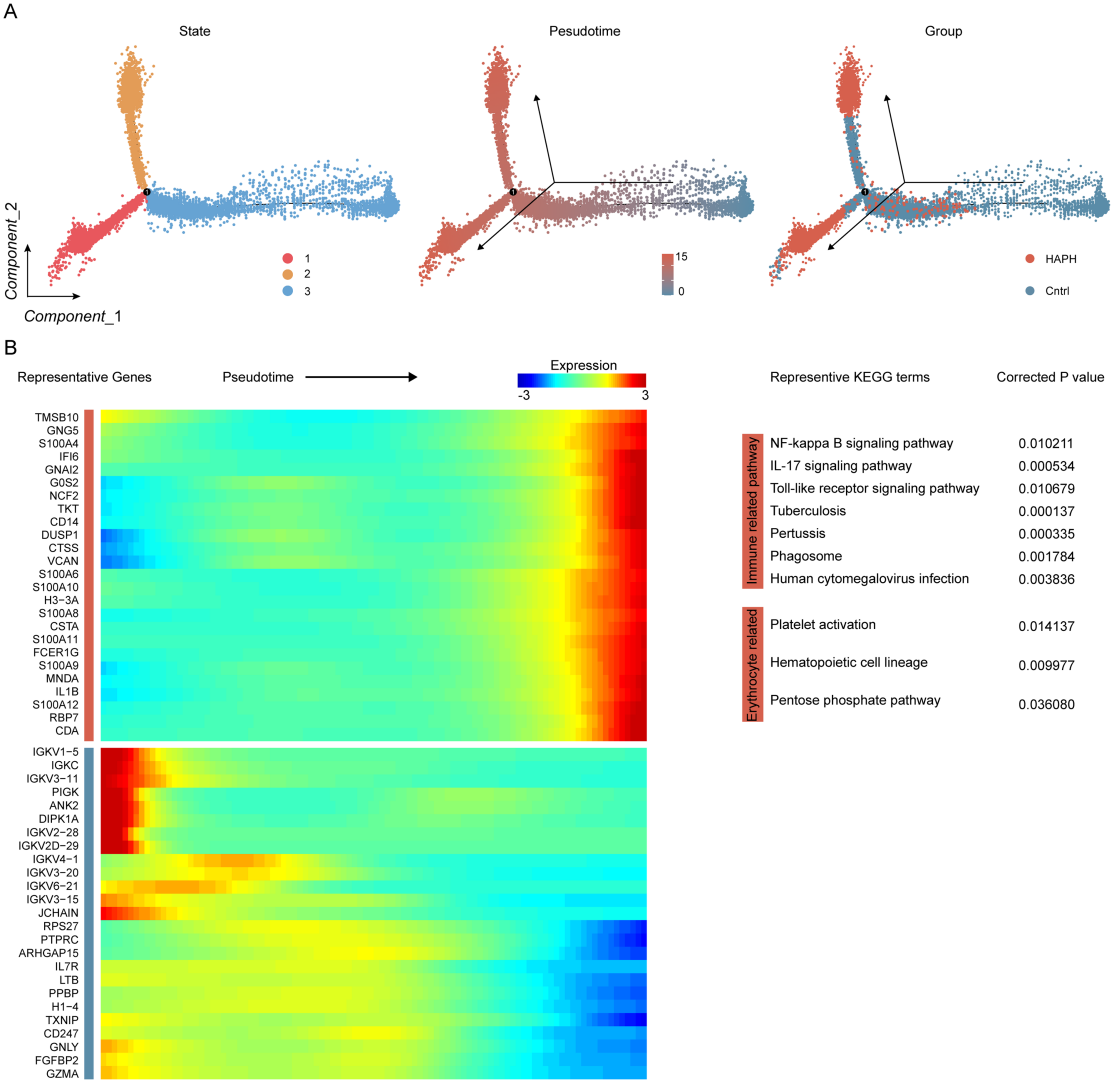
Identification of HPAH-associated genes through trajectory analysis in proximal tubule cells. **(A)** Pseudotime trajectory analysis depicts myeloid states, with color-coding indicating pseudotime progression and experimental group classification. **(B)** Heatmap visualization illustrates temporal expression dynamics, displaying genes hierarchically clustered into two discrete modules along the pseudotemporal axis. KEGG pathway enrichment analysis demonstrated statistically significant associations (FDR < 0.05).

Pseudotime trajectory analysis revealed three transcriptionally distinct states within myeloid subpopulations. The HAPH and Cntrl groups displayed divergent distribution patterns along this trajectory, with HAPH samples predominantly localized to states 1–2 and control samples concentrated in state 3 (Figure 2A). This compartmentalization correlated with state-specific transcriptional signatures. Notably, pseudotime-dependent expression profiling identified 2,615 upregulated genes associated with HAPH progression (Figure 2A; Supplementary Table S5). KEGG pathway enrichment analysis demonstrated revealed these genes were significantly enriched in immune response pathways and erythrocyte differentiation processes (Figure 2B).

### Muti-omics screened the HAPH related genes

**Figure 3:**
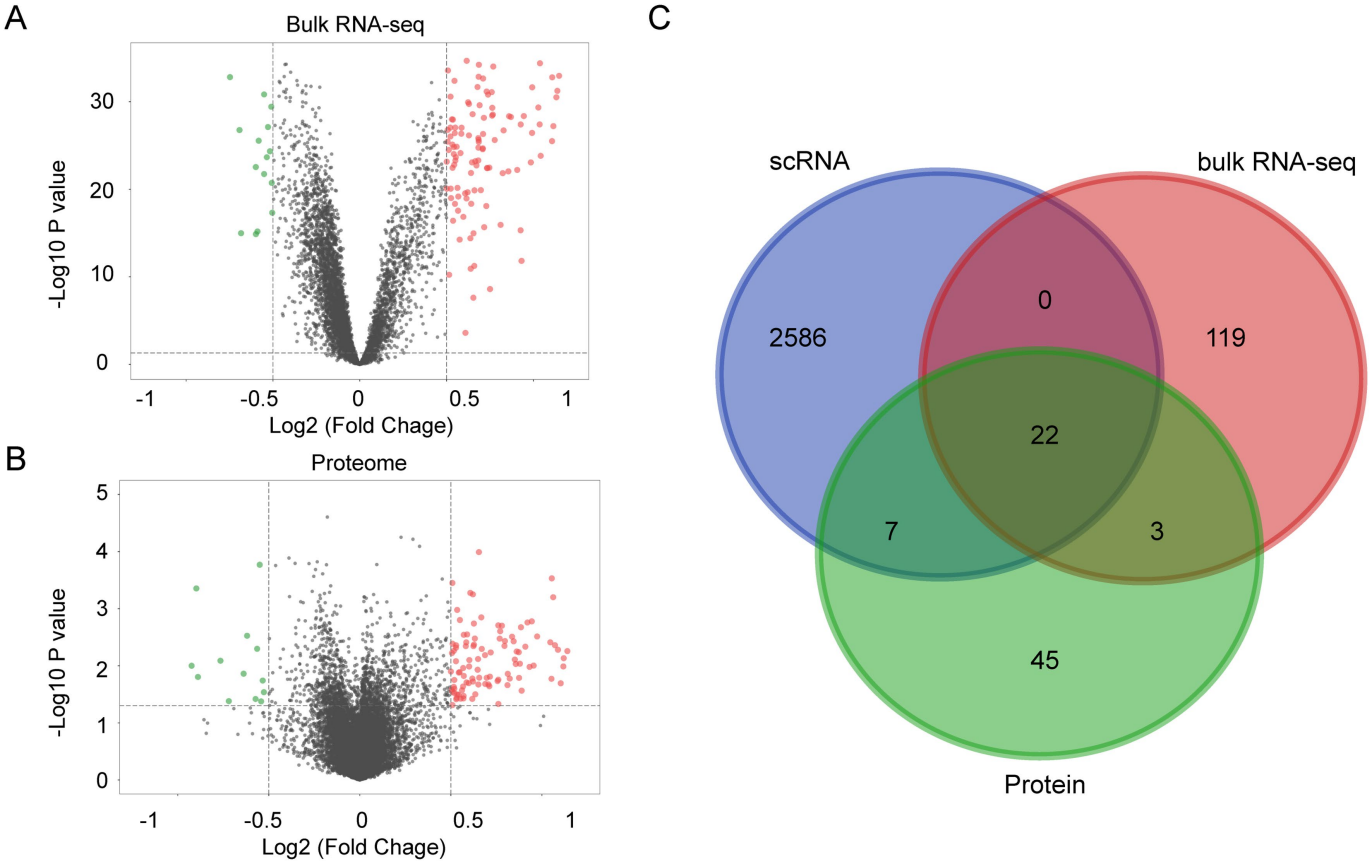
Integrated multi-omics profiling reveals HAPH-related gene signatures. Volcano plots illustrating differentially expressed genes between Control and HAPH groups from bulk RNA sequencing **(A)** and proteomic analyses **(B)**. **(C)** Venn diagram showing overlapping genes identified by multi-omics.

Differential expression analysis applied stringent thresholds (|log2 (fold change)| > 0.5, adjusted *p* < 0.05) to identify 144 significant differentially expressed genes (DEGs) using the bulk RNA-seq data (Figure 3A; Supplementary Table S6). Parallel data-independent acquisition (DIA) proteomic profiling quantified 420 and 456 plasma proteins in the HAPH and control groups, respectively. Comparative proteomic analysis revealed 77 differentially expressed proteins with |log2 (fold change)| > 0.5 and adjusted *p* < 0.05 (Figure 3B; Supplementary Table S7). Integrative multi-omics analysis demonstrated 22 consensus molecules consistently identified across scRNA-seq, bulk RNA-seq, and proteomic datasets. These overlapping biomolecules were subsequently designated as HAPH-associated signature genes (Figure 3C).

### Construction, validation, and evaluation of the HAPH diagnostic model

**Figure 4:**
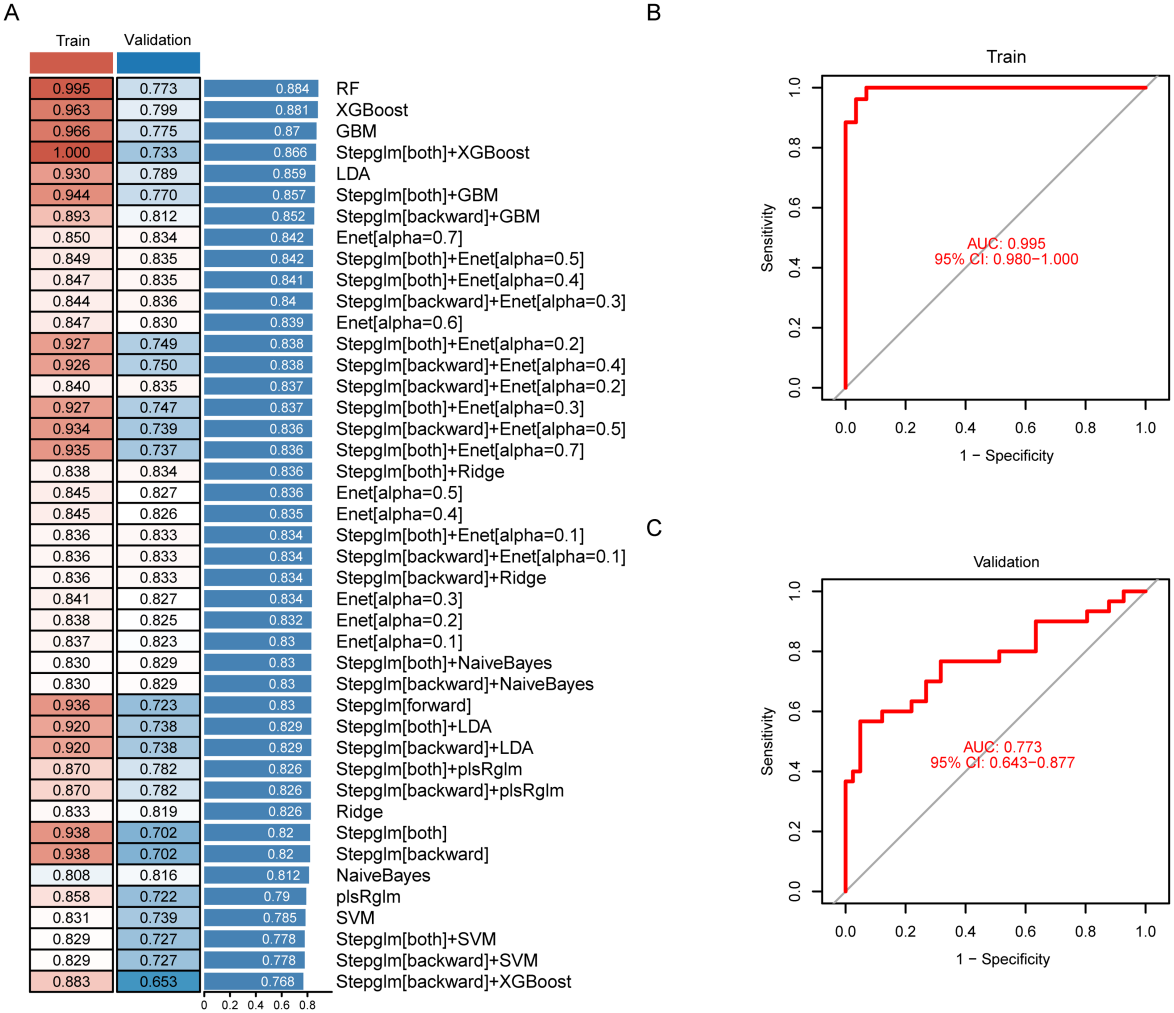
Construction, validation, and evaluation of the diagnostic model for HAPH. **(A)** The concordance index (C-index) values for different models constructed through 113 machine-learning algorithm combinations in the HAPH training set (*n* = 55) and external validation cohorts (*n* = 71). Receiver operating characteristic curves demonstrating the diagnostic performance of the HAPH signature, with AUC values reported for both the training set **(B)** and independent validation cohort **(C)**.

The 22 candidate genes identified through preliminary screening were subjected to integrated machine learning analysis to establish a diagnostic model for HAPH. Our in-house bulk RNA-seq cohort (*n* = 55) was utilized as the training set, while the external dataset (*n* = 71) served as independent validation set. Using a leave-one-out cross-validation (LOOCV) framework, we systematically evaluated 113 prediction models derived from 10 machine learning algorithms. Model performance was assessed through concordance index (C-index) evaluations across validation cohorts (Figure 4A). The random forest (RF) algorithm exhibited superior diagnostic performance, attaining the highest mean C-index (0.884). This optimized model demonstrated AUC values of 0.995 (95%CI 0.980–1.000) in the training cohort (Figure 4B) and 0.773 (95%CI 0.643–0.877) in the combined validation cohorts (Figure 4C). The Brier scores for both the training and validation sets are below 0.25, and the Hosmer-Lemeshow test *p*-value exceeds 0.05 (Supplementary Table S8), Moreover, the confusion matrix reveals accuracy rates of 0.964 (training set) and 0.704 (validation set), with both values exceeding 0.7 (Supplementary Figure S3). The final RF model incorporated six biomarker genes: HEMGN, HBG2, MYL9, ANK1, UBE2O, and RBPMS2. qPCR validation experiments confirmed significant upregulation of these biomarkers in HAPH patients relative to healthy controls (*p* < 0.05, Student’s *t*-test) (Supplementary Figure S1).

## Discussion

The expanding availability of modern transportation infrastructure has accelerated large-scale population migration to high-altitude regions, significantly increasing clinical demands for addressing HAPH (32). However, current gold-standard diagnostic methodologies remain difficult to implement in these medically underserved environments, frequently causing diagnostic delays and progressive right ventricular dysfunction (33). To bridge this gap, we constructed an advanced computational framework integrating longitudinal multi-omics profiling and ensemble machine learning models to delineate clinically actionable biomarkers with stable diagnostic trajectories.

This study elucidates the pivotal role of myeloid lineages in HAPH pathogenesis through Ro/e and contribution scoring analysis. Previous mechanistic investigations revealed that circulating myeloid cells exhibit pulmonary arteriole-specific homing, followed by differentiation into polarized pro-fibrotic macrophage subsets that drive pathological vascular remodeling (15, 34). These myeloid subgroups also exhibited functional plasticity during post-injury myocardial repair and serve as independent predictors of adverse cardiovascular outcomes (35). Building on these insights, we implemented multi-omics profiling to identify candidate biomarkers for diagnostic model construction.

This study proposed an innovative computational framework to develop a robust diagnostic signature for HAPH. After systematic evaluating 113 algorithmic combinations across 10 machine learning approaches (30, 31), we established an optimized Random Forest (RF) diagnostic model that effectively reduced dimensionality and uncovered latent pathophysiological patterns, thereby improving clinical utility. The resulting diagnostic system achieved exceptional accuracy (AUC = 0.995 in training cohorts) and maintained clinical validity (AUC = 0.773) in external validation. The validation AUC value of 0.773 exceeded the established clinical threshold (≥ 0.7), demonstrating clinical utility of the diagnostic tool. This gap was primarily attributable to inherent heterogeneity in real-world omics data and strict cohort independence measures in the validation set. While advanced computational methods have transformed pulmonary arterial hypertension (PAH) detection (36), including deep learning tools for ECG analysis and CT-based algorithms (37), their implementation in high-altitude regions remains limited by infrastructure disparities. Our innovation lied in selecting six hematological biomarkers as core diagnostic parameters. This clinically actionable biomarker panel enabled automated population-level screening while providing clinicians with an evidence-based decision support tool for early detection of HAPH. These methodological advancements could enhance therapeutic interventions and decelerate disease progression in resource-limited settings.

The selected six signatures in the RF diagnostic model included HEMGN, HBG2, MYL9, ANK1, UBE2O, and RBPMS2. Hemoglobin subunit gamma-2 (HBG2) (38) was reported in association with high-altitude pulmonary hypertension (HAPH), while RNA-binding protein with multiple splicing 2 (RBPMS2) (39) was identified as a potential biomarker and therapeutic target in idiopathic pulmonary arterial hypertension. Additionally, both Hemogen (HEMGN) (40) and Ubiquitin Conjugating Enzyme E2 O (UBE2O) (41) were implicated in oxygen transport and erythropoiesis. Furthermore, MYL9 and ANK1 were linked to vascular remodeling, including constriction. Additionally, previous literature indicates that HAPH is implicated in oxygen transport, erythropoiesis, and vascular remodeling (42, 43). Therefore, our findings were consistent with previous studies, indicating that the six HAPH-signature genes play important roles in HAPH prognosis.

While the retrospective study demonstrates promising findings, three limitations warrant consideration. Firstly, these blood biomarkers served as indicators to help identify high-risk patients who ultimately required RHC to confirm their diagnosis. Future investigations should employ standardized multicenter studies with unified protocols to comprehensively evaluate the signature’s clinical applicability. Second, although validated in independent cohorts, the predictive performance required confirmation through large-scale prospective multicenter trials. Additionally, we retain the threshold of mean pulmonary artery pressure (mPAP) ≥ 25 mmHg due to the controversial definitions of HAPH and its persistent widespread use in research literature (44, 45). Although the 2022 ESC guidelines lowered the diagnostic threshold to mPAP ≥20 mmHg for low-altitude populations, healthy high-altitude residents exhibit markedly higher baseline mPAP (approximately 10–15 mmHg) than sea-level populations (5–10 mmHg) (46). This physiological difference justifies retaining the ≥25 mmHg threshold for diagnosing HAPH in individuals living at high altitudes, despite ongoing debate.

Initially, whole blood scRNA-seq was proposed for scRNA-seq in this study. However, logistical constraints (e.g., timely transport from high-altitude collection sites >2,500 m) and technical limitations rendered this approach impractical. Specifically, whole blood was suboptimal for scRNA-seq due to abundant anucleated erythrocytes (containing minimal RNA) and fragile granulocytes prone to activation and RNA degradation during processing (47, 48). These factors introduced artifacts and compromised data quality for target immune cells. Consequently, PBMC were selected for their extended stability during cold storage, facilitating reliable transport from remote locations. Critically, PBMC encompassed key immune cell populations—including adaptive immune cells (T and B lymphocytes) and monocytes/macrophages—that orchestrated chronic inflammation and immune-mediated vascular remodeling, processes fundamental to HAPH pathogenesis (15). Nevertheless, this approach excluded granulocytes like neutrophils, precluding analysis of their role in HAPH.

The diagnosis of HAPH is confounded by comorbidity burden. To enhance diagnostic specificity and differentiate HAPH from conditions such as Chronic Mountain Sickness (CMS) and Chronic Obstructive Pulmonary Disease (COPD), we employed two key strategies. First, distinctive pathological differences were recognized: CMS centers on polycythemia whereas COPD involves airway/alveolar pathology, but HAPH was characterized by chronic hypoxia-induced pulmonary vascular remodeling. scRNA-seq leveraged these differences, revealing that monocyte-derived cells were strongly associated with HAPH pathogenesis. This observation aligned with previous studies suggesting that myeloid cells play a central role in pulmonary vascular remodeling (49, 50). Second, to ensure diagnostic precision, the training and validation cohorts were restricted to cases of isolated HAPH without comorbid cardiopulmonary conditions.

## Conclusion

In conclusion, this integrative multi-omics study systematically characterized HAPH-associated molecular biomarkers and subsequently established a machine learning-based predictive framework. These findings provided new directions and insights for the diagnosis of HAPH patients in the future.

## Data Availability

The data is provided by National Microbiology Data Center, URL is https://nmdc.cn/resource/attachment/detail/NMDCX0002155. The data presented in the study are deposited in the National Microbiology Data Center, accession number NMDCX0002155.
