# Supplementary material for: Machine learning-based integration develops an immune-derived signature for diagnosing high-altitude pulmonary hypertension
Source: Front Med (Lausanne). 2025 Sep 2;12:1603140. doi: 10.3389/fmed.2025.1603140 (PMC12436134; doi:10.3389/fmed.2025.1603140)
Supplement: Supplementary file 1 [file Supplementary_file_1.docx]

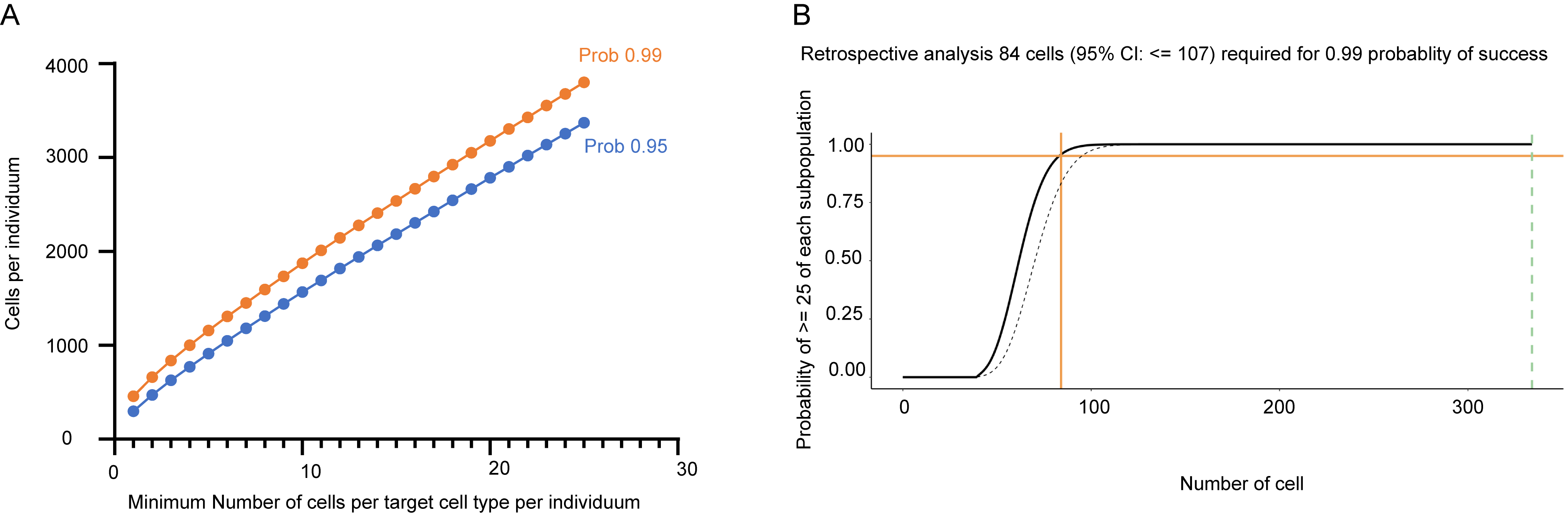


**Figure S1 Power analysis on scRNA-seq sample size.**

(A) Statistical power analysis performed using SCOPIT using both prospective and retrospective mode. Graph shows total number of cells in each individual sample (Cells per individuum) required to the minimum number of target cells in a rare cell type (population frequency 0.01 or 1%) in 1 cluster with either 0.99 or 0.95 probability of capture. (B) Graph shows the probability of capturing greater than or equal to 20 cells of a rare population with probability of 0.99 with dotted lines showing 95% confidence interval.


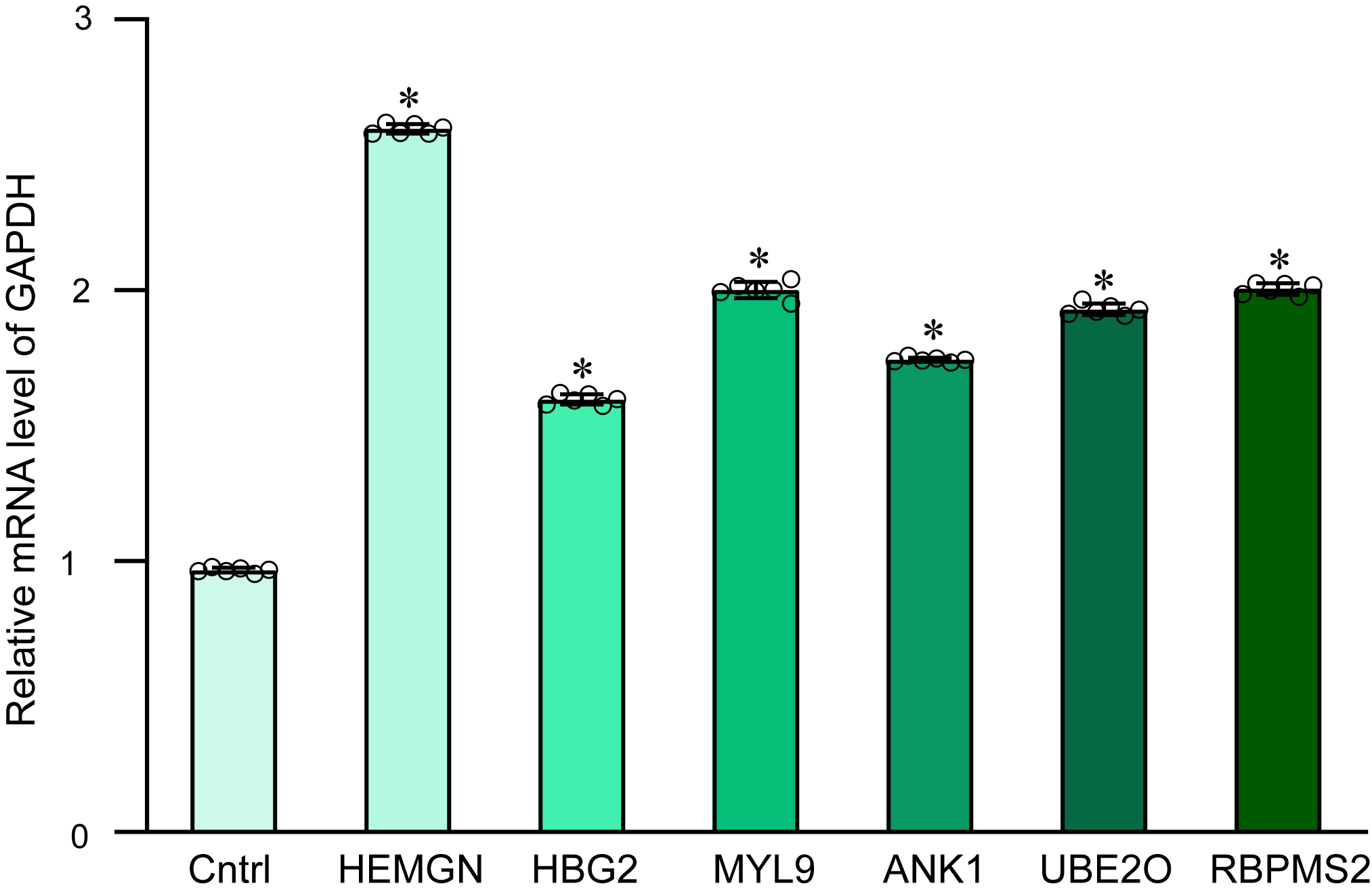


**Figure S2 Validation of HAPH-related signatures by qPCR.** The results are

presented as mean ± SD (n = 6) and were normalized to the control (*P < 0.05).


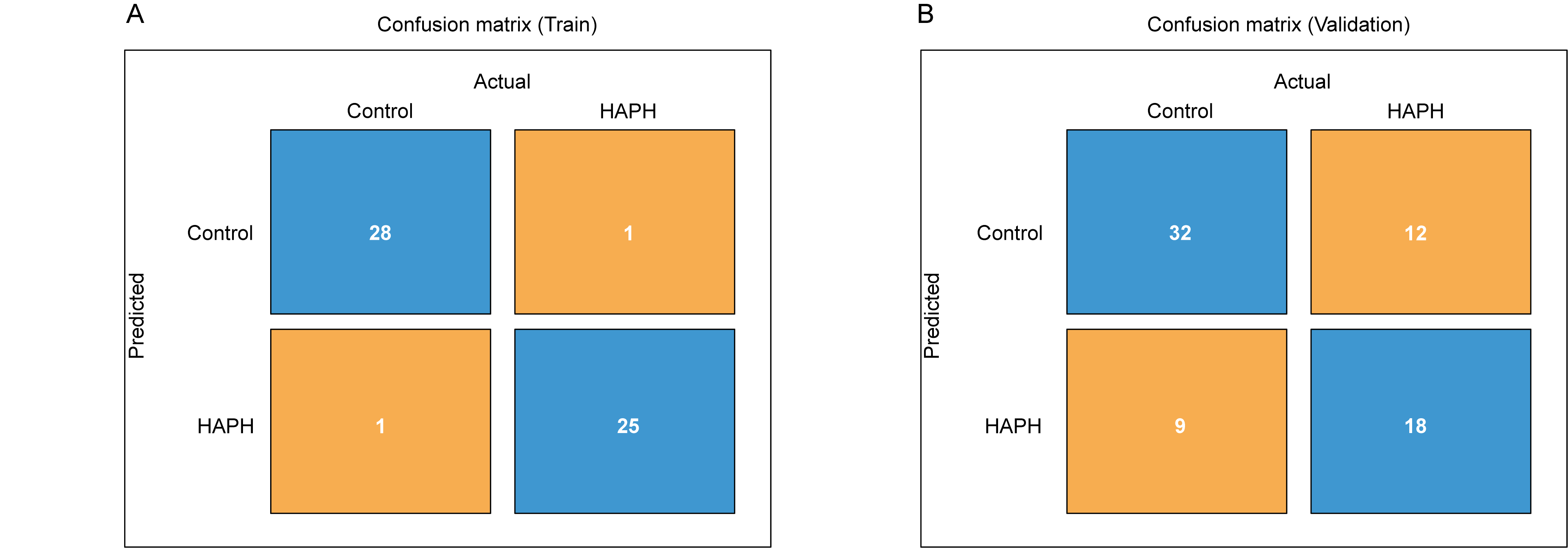


**Figure S3** **The Confusion matrix of Train (A) and Validation (B).**
